# Acknowledgement of manuscript reviewers 2014

**DOI:** 10.1186/s12933-015-0186-3

**Published:** 2015-04-15

**Authors:** Enrique Fisman, Alexander Tenenbaum

**Affiliations:** Editor-in-Chief, Cardiovascular Diabetology, Holon, Israel; Editor-in-Chief, Cardiovascular Diabetology, Tel-Aviv, Israel

## Abstract

The editors of *Cardiovascular Diabetology* would like to thank all our reviewers who have contributed to the journal in Volume 13 (2014).

**Inger-Lise Aamot**

Norway

**Monica Acevedo**

Chile

**Elmar Aigner**

Austria

**Yoshifusa Aizawa**

Japan

**Takashi Akasaka**

Japan

**Dimitrios Alexopoulos**

Greece

**Joseph Alpert**

United States of America

**Panagiotis Anagnostis**

Greece

**Martin Andreas**

Austria

**Ramaroson Andriantsitohaina**

France

**Mustafa Aparci**

Turkey

**Aleksandra Araszkiewicz**

Poland

**Johann Auer**

Austria

**Angelo Avogaro**

Italy

**Gil Bachar**

Israel

**Lina Badimon**

Spain

**Hooman Bahrami-Motlagh**

Iran

**William Bains**

United Kingdom

**Weike Bao**

United States of America

**Chris Barclay**

Australia

**Joshua Barzilay**

United States of America

**Alaaeldin Bashier**

Sudan

**Jose Bayascas**

Spain

**John Baynes**

United States of America

**Maximilian Bielohuby**

Germany

**Rafael Bitzur**

Israel

**Ellen Bohmer**

Norway

**Maria Bonsignore**

Italy

**Phillip Bowman**

United States of America

**Susan Brain**

United Kingdom

**Peter Bramlage**

Germany

**Eric Bruckert**

France

**Dennis Bruemmer**

United States of America

**Geoffrey Burnstock**

United Kingdom

**Salameh Bweir Al Dajah**

Jordan

**Christian Cadeddu**

Italy

**Lu Cai**

United States of America

**Sophie Calderari**

France

**Carmine Cardillo**

Italy

**Aleix Cases**

Spain

**Oscar Casis**

Spain

**Franco Cavalot**

Italy

**Sefa Celik**

Turkey

**Patricia Chagas**

Brazil

**Dick Chan**

Australia

**Chih-Jen Chang**

Taiwan

**Tien-Chun Chang**

Taiwan

**Nipon Chattipakorn**

Thailand

**Yi-Jen Chen**

Taiwan

**Alex Chen**

United States of America

**Jinbiao Chen**

Australia

**Shuzhen Chen**

United States of America

**Li-Ming Chen**

China

**Min Cheng**

China

**Emmanuel Chorianopoulos**

Germany

**Berit Oestergaard Christoffersen**

Denmark

**Costas Christophi**

Cyprus

**Sophie Clarke**

United Kingdom

**Kim Connelly**

Canada

**Emily Cox**

United States of America

**Alexandra M Craciun**

Romania

**Qi Dai**

United States of America

**Andreas Daiber**

Germany

**Hiroyuki Daida**

Japan

**Anindita Das**

United States of America

**Kaberi Dasgupta**

Canada

**Margie Davenport**

Canada

**Antonino De Lorenzo**

Italy

**Anthony Dear**

Australia

**Alessandra Dei Cas**

Italy

**Lea Delbridge**

Australia

**Vincent Demarco**

United States of America

**Vida Demarin**

Croatia

**Patrizia Dentelli**

Italy

**Giuseppe Derosa**

Italy

**Elio Di Segni**

Israel

**Gonzalo Diaz Soto**

Spain

**Axel Diederichsen**

Denmark

**Philipp Diehl**

Germany

**Charles Dinarello**

United States of America

**Devon Dobrosielski**

United States of America

**Helena Dominguez**

Denmark

**Massimo Donadelli**

Italy

**Marc Donath**

Switzerland

**Sami Dridi**

United States of America

**Xiao-Jun Du**

Australia

**Sergio Dubner**

Argentina

**Tobias Dünnwald**

Austria

**David Dutka**

United Kingdom

**Partha Dutta**

United States of America

**Marzena Dworacka**

Poland

**Mats Eliasson**

Sweden

**Moses Elisaf**

Greece

**Abdalla El-Mowafy**

Egypt

**Amal El-Shal**

Egypt

**Michele Emdin**

Italy

**Zivanit Ergaz**

Israel

**Jan Eriksson**

Sweden

**Julian Esparza-Romero**

Mexico

**Ricardo Jorge Esper**

Argentina

**Gian Paolo Fadini**

Italy

**Kristine Færch**

Denmark

**Abdulaziz Farooq**

Qatar

**Maria Luz Fernandez**

United States of America

**Giuseppe Fiermonte**

Italy

**Paolo Fiorina**

United States of America

**Miles Fisher**

United Kingdom

**Sabine Fredersdorf**

Germany

**Jan Magnus Fredheim**

Norway

**Tohru Fukai**

United States of America

**Yoshifumi Fukushima**

Japan

**Frédéric Fumeron**

France

**Angel Garcia-Villalon**

Spain

**Tracey Gaspari**

Australia

**Bin Geng**

China

**Pedro Geraldes**

Canada

**Sanjoy Ghosh**

Canada

**Daniel Gibbons**

United Kingdom

**Thierry Gillebert**

Belize

**Adriana Girardi**

Brazil

**Fernando Giuffrida**

Brazil

**Dulcenombre Gomez-Garre**

Spain

**Mario Gregori**

Italy

**Olaf Grisk**

Germany

**Ehud Grossman**

Israel

**Erik Grove**

Denmark

**Yong Gu**

China

**Michele Guerre-Millo**

France

**Mehran Haidari**

United States of America

**Tine Hansen**

Denmark

**Susan Harris**

United States of America

**Junichiro Hashimoto**

Japan

**Sven Haufe**

Germany

**Kevin Heffernan**

United States of America

**Simon Heller**

United Kingdom

**Aysen Helvaci**

Turkey

**Daniel Henrion**

France

**Marit Herder**

Norway

**Kjeld Hermansen**

Denmark

**Jenny Hernestal-Boman**

Sweden

**Paul Holvoet**

Belgium

**Yoshiyuki Horio**

Japan

**Keith Hruska**

United States of America

**Paul Hruz**

United States of America

**Yun Hu**

China

**Jianying Huang**

United States of America

**Yu Huang**

China

**Curtis Hughey**

United States of America

**Barbara Huisamen**

South Africa

**Kenneth Humphries**

United States of America

**Lotte Husemoen**

Denmark

**Richard Ijzerman**

Netherlands

**Toshio Imanishi**

Japan

**Catherine Ingels**

Belgium

**Esma Isenovic**

United States of America

**Nobuhiko Ishizuka**

Japan

**Edward Janus**

Bahamas

**Wei Jia**

United States of America

**Fang Fang Jiao**

Hong Kong

**Man-Hong Jim**

Hong Kong

**Manuel Jiménez-Navarro**

Spain

**Feng Jin**

United States of America

**Anna Jonsson**

Sweden

**Jens Jordan**

Germany

**Beat Jucker**

United States of America

**Paul Jurasz**

Canada

**Juulia Jylhävä**

Finland

**Yasuko K Bando**

Japan

**Takashi Kadowaki**

Japan

**Kohei Kaku**

Japan

**Thomas Karagiannis**

Greece

**Wassef Karrowni**

United States of America

**Manabu Kashiwagi**

Japan

**Zamaneh Kassiri**

Canada

**Naoto Katakami**

Japan

**Rajesh Katare**

New Zealand

**Akihiko Kato**

Japan

**Ryuichi Kawamoto**

Japan

**Takayuki Kawata**

Japan

**Jose Francisco Kerr Saraiva**

Brazil

**Zia Khan**

Canada

**Alexei Kharitonenkov**

United States of America

**Florian Kiefer**

Austria

**Kyoungkon Kim**

Korea, South

**Shokei Kim-Mitsuyama**

Japan

**Antti Kiviniemi**

Finland

**Robert Klempfner**

Israel

**Wolfgang Koenig**

Germany

**Genovefa Kolovou**

Greece

**Ping Kong**

United States of America

**Morichika Konishi**

Japan

**Grigorios Korosoglou**

Germany

**Wojciech Kosmala**

Poland

**Kazuhiko Kotani**

Japan

**Michail Koutouzis**

Greece

**Gv Krishnaveni**

India

**Yogish Kudva**

United States of America

**Shoichi Kuramitsu**

Japan

**Zenon Kyriakides**

Greece

**Nebojsa Lalic**

Serbia

**Regis Lamberts**

New Zealand

**Xiaopeng Lan**

China

**Steve Lancel**

France

**Chim Lang**

United Kingdom

**Roberto Latini**

Italy

**Chiara Lazzeri**

Italy

**Djamel Lebeche**

United States of America

**Erwan Leclair**

Canada

**Natalie Lefort**

Canada

**Michael Lehrke**

Germany

**Yuchi Li**

United States of America

**Vassilios Liakopoulos**

Greece

**Qiangrong Liang**

United States of America

**Jou-Wei Lin**

Taiwan

**Shing-Jong Lin**

Taiwan

**Jianmin Liu**

United States of America

**Wenyu Liu**

United States of America

**Naifeng Liu**

China

**Milos Ljubisavljevic**

United Arab Emirates

**Denis Loiselle**

New Zealand

**Oscar Lorenzo**

Spain

**Cecilia Low Wang**

United States of America

**Jun Lu**

New Zealand

**Annika Lundström**

Sweden

**Rong Luo**

United States of America

**Rogelio Alberto Machado**

Argentina

**Paolo Madeddu**

United Kingdom

**Norikazu Maeda**

Japan

**Namita Mahalle**

India

**Laura Beatriz Maifrino**

Brazil

**Ville-Petteri Mäkinen**

Finland

**Aline Marcadenti**

Brazil

**M. Carmen Martinez**

France

**Luis Masana**

Spain

**Filipa Mascarenhas-Melo**

Portugal

**Walter Masson**

Argentina

**Satoaki Matoba**

Japan

**Masafumi Matsuda**

Japan

**Takayuki Matsumoto**

Japan

**Takeshi Matsumura**

Japan

**Carrie Mcadam-Marx**

United States of America

**Maralee Mcvean**

United States of America

**Kimberley Mellor**

New Zealand

**Lampros Michalis**

Greece

**Takayuki Miki**

Japan

**Ivana Mikolasevic**

Croatia

**Shin-Ichiro Miura**

Japan

**Toru Miyoshi**

Japan

**Børge Moe**

Norway

**Hamid Moha Ou Maati**

France

**Manuel Monreal**

Spain

**Hamid Moradi**

United States of America

**Evangelia Mourmoura**

France

**Elza Muscelli**

Brazil

**Laszlo Nagy**

Hungary

**Hironori Nakagami**

Japan

**Katsuyuki Nakajima**

Japan

**Kazufumi Nakamura**

Japan

**Vihang Narkar**

United States of America

**Shyh-Chang Ng**

Singapore

**Gregory Nichols**

United States of America

**Yoshihiko Nishio**

Japan

**Hitoshi Nishizawa**

Japan

**Yoko Noda**

Japan

**Atsushi Nohara**

Japan

**Njord Nordstrand**

Norway

**Salvatore Novo**

Italy

**Hossein Noyan**

Canada

**Tsuyoshi Nozue**

Japan

**Shigeharu Numao**

Japan

**Eiji Oda**

Japan

**Martha O'Donnell**

United States of America

**Anne Pernille Ofstad**

Norway

**Tsuyoshi Ohkura**

Japan

**Koichi Ojima**

Japan

**Katashi Okoshi**

Brazil

**Marina Okoshi**

Brazil

**Silvio Assis Oliveira Junior**

Brazil

**Christine Oltman**

United States of America

**Altan Onat**

Turkey

**Kwok Leung Ong**

Australia

**Wayne Orr**

United States of America

**Alberto Ortiz**

Spain

**Oleg Osadchii**

Denmark

**Karen O'Shea**

United States of America

**Horacio Osorio**

Mexico

**Alfonso Otero Gonzalez**

Spain

**Margriet Ouwens**

Germany

**Jun-Ichi Oyama**

Japan

**Lucia Pacifico**

Italy

**Jisun Paik**

United States of America

**Bradley Palmer**

United States of America

**Demosthenes Panagiotakos**

Greece

**Nikolaos Papanas**

Greece

**Tiziana Parasassi**

Italy

**Anthony Passerini**

United States of America

**Hemang Patel**

United States of America

**Himanshu Pathak**

United States of America

**James Pearson**

Australia

**Thierry Pedrazzini**

Switzerland

**Chris Pemberton**

New Zealand

**Yu-Sen Peng**

Taiwan

**Frederik Persson**

Denmark

**Jonas Persson**

Sweden

**Wu Ping**

United States of America

**Anna Polesskaya**

France

**Karen E Porter**

United Kingdom

**Marek Postula**

Poland

**Rudolf Prager**

Austria

**David Preiss**

United Kingdom

**Giuseppe Pugliese**

Italy

**Lu Qi**

United States of America

**Vasudevan Ramachandran**

Malaysia

**Sivakumar Ramalingam**

India

**Susana Ravassa**

Spain

**Zeljko Reiner**

Croatia

**Flavio Reis**

Portugal

**Jun Ren**

United States of America

**Graham Rena**

United Kingdom

**Miriam Ribeiro**

Brazil

**Oliver Rider**

United Kingdom

**Nasser Rizk**

Qatar

**Ali Rizvi**

United States of America

**Manfredi Rizzo**

Italy

**Milagros Rocha**

Spain

**Luc Rochette**

France

**Bruno Rodrigues**

Brazil

**Andrea Rosanoff**

United States of America

**Jaya Rosenmeier**

Denmark

**Ina-Maria Rückert**

Germany

**Junichi Sadoshima**

United States of America

**Soichi Sakai**

Japan

**Juha Saltevo**

Finland

**Rafael Salto**

Spain

**Javier Salvador**

Spain

**Matej Samos**

Slovakia

**Gaetano Santulli**

United States of America

**Vijaya Sarathi**

India

**Masataka Sata**

Japan

**Simone Scarlata**

Italy

**Thomas Scheeren**

Netherlands

**Guntram Schernthaner**

Austria

**Thomas Schindler**

United States of America

**Georg Schlieper**

Germany

**Jochen Schneider**

Luxembourg

**Eszter Sélley**

Hungary

**Amir Houshang Shemirani**

Hungary

**Katsumi Shibata**

Japan

**Michio Shimabukuro**

Japan

**Latika Sibal**

United Kingdom

**Agnieszka Siejka**

Poland

**Felipe Simon**

Chile

**Robert Sladek**

Canada

**Jessica Smith**

United States of America

**Wayne Smith**

South Africa

**Janet Snell-Bergeon**

United States of America

**Harald Sourij**

Austria

**Ian Spence**

Australia

**Eberhard Standl**

Germany

**Norbert Stefan**

Germany

**Ki Chul Sung**

Korea, South

**Andrzej Surdacki**

Poland

**Chrysanthos Symeonidis**

Greece

**J David Symons**

United States of America

**Marijana Tadic**

Serbia

**Heinrich Taegtmeyer**

United States of America

**Yasuo Takahashi**

Japan

**Noboru Takamura**

Japan

**Sevda Tanrikulu Kucuk**

Turkey

**Marianne Tare**

Australia

**Lise Tarnow**

Denmark

**Sima Tarzami**

United States of America

**Ashraf Taye**

Egypt

**Chunbo Teng**

China

**Nicholas Tentolouris**

Greece

**Yasuo Terauchi**

Japan

**Dimitris Tousoulis**

Greece

**Al Tramontano**

United States of America

**Hung-Fat Tse**

Hong Kong

**Johnathan Tune**

United States of America

**Mariko Uehara**

Japan

**Anetta Undas**

Poland

**Jaap Van Buul**

Netherlands

**Charissa Van Den Brom**

Netherlands

**Roberto Vazquez-Padron**

United States of America

**Saula Vigili De Kreutzenberg**

Italy

**Manlio Vinciguerra**

United Kingdom

**Antony Vinh**

Australia

**Chris Vlahos**

United States of America

**Jan Von Der Thüsen**

Netherlands

**Jun Wada**

Japan

**Christian Wahl-Schott**

Germany

**Cherry Wainwright**

United Kingdom

**Daowen Wang**

China

**Huishan Wang**

China

**Ping Wang**

United States of America

**Rui Wang**

Canada

**Shaobin Wang**

United States of America

**Tzung-Dau Wang**

Taiwan

**Wei Wang**

United States of America

**Ying Wang**

United States of America

**Marie-Louise Ward**

New Zealand

**Dorohtee Weihrauch**

United States of America

**István Wittmann**

Hungary

**Jeong-Taek Woo**

Korea, South

**Li-Ling Wu**

China

**Yen-Wen Wu**

Taiwan

**Ying Wu**

United States of America

**Morten Würtz**

Denmark

**Haipeng Xiao**

China

**Da Xing**

China

**Aimin Xu**

Hong Kong

**Biao Xu**

China

**Xihui Xu**

United States of America

**Hideya Yamamoto**

Japan

**Wenying Yang**

China

**Yuichiro Yano**

United States of America

**Yung-Hsin Yeh**

Taiwan

**Derek Yellon**

United Kingdom

**Utako Yokoyama**

Japan

**Xi-Yong Yu**

China

**Guoyue Yuan**

China

**Hasniza Zaman Huri**

Malaysia

**Christine Zelenak**

Canada

**Sami Zguira**

France

**Ji-Xiang Zhang**

China

**Shaoping Zhang**

New Zealand

**Shaojie Zhang**

United States of America

**Xiaodong Zhang**

United States of America

**Jiajun Zhao**

China

**Ting Zhao**

United States of America

**Guangzhen Zhong**

China

**Hao Zhu**

United States of America

**Minmin Zhu**

China

**Muhammad Zia-Ul-Haq**

Pakistan

**Tali Zitman-Gal**

Israel

